# HIV-free survival according to the early infant-feeding practices; a retrospective study in an anti-retroviral therapy programme in Makurdi, Nigeria

**DOI:** 10.1186/s12879-015-0871-6

**Published:** 2015-03-18

**Authors:** Emmanuel A Anígilájé, Othniel J Dabit, Ayodotun Olutola, Bem Ageda, Sunday A Aderibigbe

**Affiliations:** Department of Paediatrics, Benue State University, Makurdi, Benue State Nigeria; Centre for Clinical Care and Clinical Research, 29 Mambilla Street, Off Aso Drive, Maitama, Abuja, Nigeria; Department of Obstetrics and Gynaecology, Federal Medical Centre, Makurdi, Benue State Nigeria; Department of Community Medicine, University of Ilorin, Ilorin, Kwara State Nigeria

**Keywords:** HIV-exposed infants, HIV-free survival, Early-infant feeding practice, Makurdi, Nigeria

## Abstract

**Background:**

In Nigeria, reports of the outcomes of prevention of mother to child transmission of HIV (PMTCT) interventions had been limited to the MTCT rates of HIV, with no information on HIV-free survival (HFS) in the HIV-exposed infants over time.

**Methods:**

A retrospective study between June 2008 and December 2011 at the Federal Medical Centre, Makurdi, Nigeria comparing HFS rates at 3 and 18 months according to the infant feeding pattern at the 6th week of life. HFS was assessed by Kaplan-Meier analysis and association of maternal and infant variables and risk of HIV acquisition or death was tested in a Cox regression analysis.

**Results:**

801 HIV uninfected infants at 6 weeks of life were studied in accordance with their reported cumulative feeding pattern. This includes 196 infants on exclusive breast feeding (EBF); 544 on exclusive breast milk substitute (EBMS) feeding and 61 on mixed feeding (MF). The overall HFS was 94.4% at 3 months and this declined significantly to 87.1% at the 18 months of age (p-value = 0.000). The infants on MF had the lowest HFS rates of 75.7% at 3 months and 69.8% at 18 months. The HFS rate for infants on EBF was 97.4% at 3 months and 92.5% at 18 month whilst infants on EBMS had HFS of 99.1% at 3 months and 86.2% at 18 months. A higher and significant drop off in HFS at the two time points occurred between infants on EBMS (12.9%) compared to infants on EBF (4.9%), p-value of 0.002, but not between infants on MF (5.9%) and EBMS, p-value of 0.114 and those on MF and EBF, p-value of 0.758. In Cox regression multivariate analyses; MF, gestational age of ˂ 37 weeks, and a high pre-delivery maternal viral load were consistently associated with HIV infection or death at 3 months and 18 months (p ˂0.05).

**Conclusion:**

For a better HFS in our setting; MF must be avoided, efforts to deliver babies at term in mothers with reduced viral load are advocated and EBF must be promoted as the safest and the most feasible mode of infant-feeding.

## Background

Among the few mothers that accessed prevention of mother to child transmission of HIV (PMTCT) services in Nigeria, the reports of successes in interventions had been limited to stating the MTCT rates of HIV, indicating values in the range of 1.1%-11%, over 6 weeks to 18 months of age [1-5]. However, limited information exists on the longitudinal follow-up and survival of these HIV-exposed infants.

In 2010, the World Health Organization (WHO) proposed HIV-free survival as the gold standard for evaluating National PMTCT programme effectiveness [6]. HIV-free survival captures information on the direct effects and the indirect effects of PMTCT programmes. The direct effects include information on HIV infection at birth and through breastfeeding and the deaths prevented [6]. The indirect survival benefits that may accrue to HIV-exposed children who do not become infected are also important [6].

Breastfeeding provides immunity against diarrhoeal disease and pneumonia, but these benefits have to be balanced against the risk of HIV transmission via breast milk [7,8]. Replacement feeding when exclusive is expected to prevent HIV transmission that occurs via breast milk. However, in areas where water supply is unsafe and poverty is rife, it increases infants’ mortality from diarrhoea and malnutrition [9]. Therefore, the WHO recommends replacement feeding as an alternative to breastfeeding only when it is affordable, feasible, acceptable, safe and sustainable (AFASS) [10]. Unfortunately, in Nigeria, the AFASS criteria are rarely met even in programmes where Breast-Milk Substitutes (BMS) are provided free of cost and mixed feeding is a common practice [11,12]. Mixed feeding combines the risk of HIV transmission through breastfeeding with the increased risk of mortality associated with replacement feeding [13,14].

Bearing the foregoing in mind and to the best of the Authors’ knowledge, there is no study that has reported the HIV-free survival rates in Nigerian HIV-exposed infants.

Therefore, we conducted a retrospective study among HIV-exposed babies delivered between June 2008 and December 2011 and compared the cumulative HIV-free survival rates at 3 and 18 months of life according to the early infant feeding modalities at the 6th week of life.

## Methods

### Study area and setting

The study was carried out at the Paediatric antiretroviral therapy (ART) Clinic of the Riverside Specialist Clinics of the FMC, Makurdi. The facility is supported by the AIDS Prevention Initiative in Nigeria (APIN)/Harvard PEPFAR (The USA President’s Emergency Plan for AIDS Relief) programme.

### Ethical consideration

Upon recruitment into the care and ART programme, parents or caregivers of the HIV-exposed infants had initially provided written informed consent for the use of their data for research as approved by the Research and Ethics Committee of the FMC, Makurdi and the APIN/Harvard PEPFAR programme. For this study, permission was sought for and gotten for the use of the relevant data.

### Study design and population

It was a retrospective study between June 2008 and December 2011. Included were HIV-exposed uninfected infants at 6 weeks that were delivered to HIV-infected mothers who had received PMTCT interventions at FMC, Makurdi. Excluded were; (i) infants of HIV-infected mothers who did not attend antenatal care at FMC, Makurdi and whose mothers did not receive antiretroviral drugs (ARVs) for PMTCT;(ii) infants who had received blood transfusion and or those with a proven history of sexual abuse that may suggest horizontal mode of HIV transmission; (iii) infants who were on breastfeeding but whose mothers died before the 6th week of life; (iv) the second of a set of twin; (v) infants whose DNA/PCR results were equivocal (vi); and infants whose DNA/PCR were positive at the 6th week of life that may be suggestive of an in-utero or intra-partum HIV transmission [15,16].

### Follow-up of HIV positive pregnant mothers and their infants and operational definitions

HIV-exposed infants were first seen within the first 24 hours of life and thereafter, every 2-week for the first 6 weeks, every month for the first 3 months and 3-monthly till the age of 18 months. Early infant diagnoses with the DNA/PCR were offered routinely at the 6th week and at the 3rd month of life and the HIV antibody test at the 18th month when infants were then discontinued from care when found to be HIV uninfected and in optimal nutritional state. Infants found to be HIV-infected at any point in time were promptly commenced on highly active antiretroviral therapy (HAART) in accordance with the clinical and age-dependent immunological criteria of WHO guideline of 2006 [17].

Infants were regarded as HIV-infected if two samples were positive for HIV DNA/PCR and or there was a positive HIV serology at 18 months. If HIV DNA/PCR test was negative and or HIV serology was negative at the 18 months, the infants were said to be HIV uninfected. Co-trimoxazole prophylaxis was given to all infants from the 6th week of life. HIV infected women who had been on HAART before conceptions were counselled on optimal adherence to HAART throughout the antenatal visits. PMTCT treatment guidelines for the HIV-infected pregnant women also followed the 2006 WHO recommendations [17]. During the first ANC visit, pregnant women whose HIV status were unknown received HIV testing and counselling and those found to be HIV-infected were screened for clinical and immunological eligibility for the commencement of HAART. Mothers who were eligible [i.e., having Cluster-of- differentiation- 4 T lymphocytes (CD4) counts ˂ 200 cells/mm^3^] received HAART regimen including Zidovudine (AZT) or Stavudine (d4T) plus Lamivudine (3TC) plus Nevirapine (NVP). Women not eligible for HAART had AZT from 28 weeks of gestation and were given single-dose NVP and 3TC during labour and delivery and also, a 7-day tail of 3TC and AZT postpartum. HIV-exposed babies received a single dose of Nevirapine (sdNVP) which was given within the first 72 hours of life and also AZT given for 7 days. Babies have option of being fed with either exclusive breast milk substitutes feeding (EBMS) for the first 6 months of life when AFASS or exclusive breastfeeding (EBF) for the first 6 months of life in accordance with the Nigerian Guidelines for Paediatric HIV and AIDS Treatment and Care of 2007 [18]. Mixed feeding was thoroughly discouraged. Mothers received infant-feeding counselling during antenatal care and were allowed to make informed choices on EBMS or EBF. For mothers who opted for EBMS, they were taught on the hygienic technique of constituting the BMS using cooled boiled water, stipulating the use of cups and discouraging bottle-feeding. The programme provided a 6-month (on a monthly discrete prescription) supply of free infant formula to mothers who opted for EBMS. In order to enhance the safety of EBMS, mothers also received free Sodium hypochlorite (Water Guard®). Detailed feeding histories were from maternal recalls.

For the purpose of this study, the cumulative feeding pattern reported up to the 6th week of life was used to group the infants. The definitions of EBF, EBMS (Replacement feeding) and Mixed feeding (MF) were as described previously [7,17,19]. Two episodes of instances of mixed feeding were required for MF in this study. Late-post-natal- HIV- transmission (PNT) was assumed in infants who were DNA/PCR negative at their 6-week visit but were DNA/PCR positive at the 3rd month or HIV serology positive subsequently. This reflects HIV infection that is unequivocally attributable to breastfeeding [7,15] having excluded infants with horizontal HIV transmission and this was the focus of this study.

### Data collection

Data were from both electronic databases and Patients’ Record files of mother-infants pairs. Time-dependent information such as; infants’ HIV status state (positive or negative), infants that were Lost to follow-up, and the Vital status state (dead or alive) were particularly sought for cumulatively up to the 6th week, the 3rd month and the 18th month of follow-up.

### Measured outcome

Cumulative HIV- free survival rates at 3 and 18 months of follow-up according to early infants’ feeding at 6 weeks of life.

### Statistical analysis

Statistical analysis was done using the SPSS version 16. Characteristics were summarized using means and medians for continuous variables and proportions for categorical variables. The Mann–Whitney (two groups) and Kruskal-Wallis (three groups) tests were used for association between and among the medians and unpaired ‘t’ test (two groups) and one-way ANOVA (three groups) were used for the means. Pearson Chi-squared test or Fischer’s exact test was used for categorical variables. Cumulative HIV- free survival, HIV transmission and Mortality at the 3rd and 18th month of life were assessed by Kaplan-Meier analysis according to the reported early infant feeding pattern (i.e. EBF, EBMS, and Mixed) at the 6th week. Sample *t*-test between proportions was used to test the decay in HFS rates between EBF and EMBS over 3 to 18 months. Association of maternal and infant variables and risk of HIV acquisition or death was tested in a Cox regression analysis. The model included maternal and infant factors significant at p ≤ 0.1 in Univariate analyses and *a priori* factors [16]-maternal CD4 count and viral load, breast mastitis, low birth weight, oral sores/oral thrush in the infants- for MTCT regardless of their level of significance. The clinical endpoints for calculating HIV-free survival included time to HIV infection or time to death. The date of the first event (either HIV infection or death if the child experienced both events) was used as the event date. Censoring for the HIV-free survival, HIV-infection and mortality outcomes occurred due to loss –to- follow-up at the visit when this was first noted. For all analyses, confidence intervals (CI) were set at 95% level and p-values less than 0.05 were considered statistically significant.

## Results

**Figure 1:**
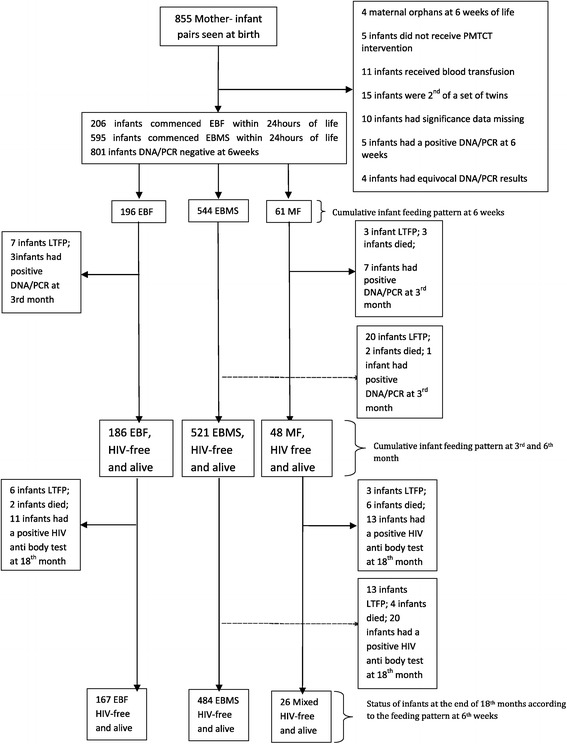
**Schematic diagram of the follow-up of the infants in the study.** Legends: LTFP: loss to follow up; EBF: exclusive breast feeding; EBMS: exclusive breast milk substitute feeding; MF: mixed feeding; PMTCT; prevention of mother to child transmission of HIV.

Figure 1 is the schematic diagram of the follow-up of the infants in the study. 855 infants were born alive to HIV-infected mothers but only 801 infants who were DNA/PCR negative at the 6th week were included in the study. Reasons for the exclusion of the other 54 infants were as shown in Figure 1. Among these 801 infants, 206 mothers had initially opted for EBF and 595 mothers for EBMS within the first 24 hours of birth (i.e., intention -to -feed). However, by the 6th week of follow-up, 61 infants were already mixed- fed. This included 10 of the initial exclusive breast-feeders (i.e., EBF uptake of 95.1%) and 51 of the initial replacement feeders (EBMS uptake of 91.4%). Among the MF infants, the infant feeds in addition to the mix of breast milk/BMS (43 infants, 70.5%) were maize gruels in 12 infants, (19.6%, 12/61) and cocoa beverages (Milo®) in 6 infants, (9.8%, 6/61). At the 3rd month of life, after excluding infants that were lost to follow-up and those that died, 94.9% (186/196) of the infants were still on EBF, 95.8% of the infants were still on EBMS, and 78.7% were still mix-feeding. Same proportions were on these feeding patterns by the 6th month.

### Maternal and infant characteristics according to the early infants’ feeding pattern

Table 1 shows that out of the 801 infants, 401 were males (M) and 400 were females (F) with a M; F ratio of 1: 0.99. Ninety four percent (751/801) of the mothers in this cohort received HAART for their own HIV disease. Most of these women (336/751, 44.7%) were on HAART before pregnancy. Among the 415 mothers who initiated HAART during pregnancy, 290 (38.6%) were commenced during the first trimester while 25 (3.3%) and 100 (13.3%) initiated HAART during the second and the third trimester respectively. A significant number of mothers who opted for EBMS were primiparous when compared with EBF alone (P value 0.008) as well as when compared with infants on EBF and MF (P value 0.04).

**Table 1:** **Maternal and infant characteristics according to the early (first 6 weeks) feeding pattern**

**Variables**	**EBF N (%)**	**EBMS N (%)**	**MF N (%)**
Gender			
Male	95 (48.5)	278 (51.1)	28 (45.9)
Female	101 (51.5)	266 (48.9)	33 (54.1)
Total number	196	544	61
Birth weight			
Mean (SD)	2.91 (0.35)	2.88 (0.43)	2.86 (0.36)
˂2500 g	17 (8.7)	59 (10.8)	4 (6.6)
≥2500 g	179 (91.3)	485 (89.2)	57 (93.4)
Gestational age at birth			
˂37 weeks	5 (2.6)	21 (3.9)	2 (3.3)
≥37 weeks	191 (97.4)	523 (96.1)	59 (96.7)
Mode of delivery			
Vaginal	189 (96.4)	513 (94.3)	59 (96.8)
EME C/S	6 (3.1)	27 (5.0)	1 (1.6)
ELE C/S	1 (0.5)	4 (0.7)	1 (1.6)
ROM			
˂4 hours	182 (92.9)	507 (93.2)	57(93.4)
≥4 hours	13 (6.6)	33 (6.1)	3 (4.9)
No ROM	1(0.5)	4 (0.7)	1 (1.6)
Vaginal tear			
Yes	50 (25.5)	133(20.8)	17 (27.9)
No	146 (74.5)	431 (79.2)	44 (72.1)
Infants’ oral thrush			
Yes	3 (1.5)	10 (1.8)	3 (4.9)
No	193 (98.5)	534(98.2)	58 (95.1)
Infants’ oral sore			
Yes	5 (2.6)	8 (1.5)	2 (3.3)
No	191 (97.4)	536 (98.5)	59 (96.7)
Mother’s age group in years			
Mean (SD)	28.8 (5.4)	29.0(5.4)	28.9(5.6)
15 – 24	38 (19.4)	99 (18.2)	13 (21.3)
25 - 34	133 (67.9)	360 (66.2)	40 (65.6)
35 - 39	21 (10.7)	71 (13.1)	5 (8.2)
≥40	4 (2.0)	14 (2.6)	3 (4.9)
*Mothers’ CD4 count			
(Cell/mm^3^)	176	490	55
Median (IQR)	408 (335–537.8)	403 (316–557)	377 (286–505)
˂ 200	10 (5.1)	39 (7.2)	6 (9.8)
200 – 349	41 (20.9)	119 (21.9)	13 (21.3)
359 – 499	79 (40.3)	178 (32.7)	21 (34.4)
≥500	46 (23.5)	154 (28.3)	15 (24.6)
Missing	20 (10.2)	54 (9.9)	6 (9.8)
*Mothers’ viral load			
(Copies/ml)	175	474	56
Median (IQR)	200 (200–2198)	200 (200–1468.8)	200 (200–2775)
˂ 1000	117(59.7)	328 (60.3)	39 (63.9)
1000 – 9999	33 (16.8)	95 (17.5)	9 (14.8)
10,000 – 99, 999	19 (9.7)	41 (7.5)	6 (9.8)
≥100, 000	6 (3.1)	10 (1.8)	2 (3.3)
Missing	21 (10.7)	70 (12.9)	5 (8.2)
^PMTCT Intervention in the mother			
HAART	188 (95.9)	505 (92.8)	58 (95.1)
ARV	8 (4.1)	39 (7.2)	3 4.9
*Mothers’ hemoglobin			
˂10 g/dl	21 (10.7)	86 (15.8)	11 (18.0)
≥10 g/dl	175 (89.3)	458 (84.2)	50 (82.0)
§Mothers’ Parity			
1	84(42.9)	263 (48.3)	28 (45.9)
2 – 4	81 (41.3)	237 (43.6)	28 (45.9)
≥5	31 (15.8)	44 8.1	5 8.2)
Mothers’ religion	188(95.9)	528 (97.1)	60 (98.4)
Christianity	8 (4.1)	16 (8.1)	1 (1.6)
Islam			
Breast problems reported or diagnosed at any time between birth and the 6th month			
Yes	4 (2.0)	14(2.6)	2 93.3)
No	196(98.0)	530 (97.4)	59 (96.7)
Mothers’ vital status at 18 month			
Dead	2 (1.0)	5 (0.9)	0 (0)
Alive	194 (99.0)	539 (99.1)	61 (100)
Mothers’ level of education			
None	52 (26.5)	109 (20.0)	13 (21.3)
Primary	44 (22.4)	133 (24.4)	12 (19.7)
Secondary	97 (49.5)	296 (54.4)	36 (59.0)
Tertiary	3 (1.5)	6 (1.1)	0 (0)
Mothers’ marital status			
Without a partner	7 (3.6)	29 (5.3)	5 (8.2)
With a partner	189 (96.4)	515 (94.7)	56 (91.8)
Mothers/family monthly income			
Below minimum wage (~105 USD)	149 (76.0)	409 (75.2)	52 (85.2)
Minimum wage and above	47 (24.0)	125 (24.8)	9 (14.8)

**Legends**: N = number; SD = standard deviation; IQR = interquartile range; EME C/S = emergency caesarean section; ELE C/S = elective caesarean section; EBF = exclusive breast feeding; EBMS = exclusive breast milk substitute feeding; MF = mixed feeding; ROM = rupture of membrane; PMTCT = prevention of mother to child transmission of HIV; USD = united states dollars; * = pre-delivery values; ^= Most of these women (336) were already on HAART before conception, 290 were commenced on HAART at the first trimester and 25 and 100 mothers started HAART during the second and the third trimester respectively;§ = significant p values exist among primiparous women who opted for EBMS when compared with EBF alone (P value 0.008) as well as when compared with infants on EBF and MF (P value 0.04).

### HIV-free survival, HIV-infection and infants’ mortality according to the early infants’ feeding pattern

Table 2 shows that the overall cumulative HIV-free survival rate was 94.4% at 3 months and this declined significantly to 87.1% at the 18 months of age (p-value; 0.000). The 3 month cumulative HIV-free survival rates for the EBF, the EBMS and the Mixed fed infants were 97.4%, 99.1%, and 75.7% respectively. Pair-wise comparison at the 3rd month for HIV-free survival rates between EBF and EBMS infants was not significant at p-value of 0.078 but was significant between EBF and MF (p-value; 0.000) and between EBMS and MF (p value; 0.000). There was a fall in the cumulative HIV-free survival rates at 18 month from the 3rd month rates for all the three feeding patterns with values for EBF, EBMS and MF being 92.5%, 86.2% and 69.8% respectively. When the rate of fall in HFS between the two time points was compared between infants on EBF (97.4% to 92.5%, i.e. 4.9%) and those on EBMS (99.1% to 86.2%, 12.9%), a significant difference (p-value of 0.002) was gotten. The rate of fall in HFS was however not significant between infants on MF (75.7% to 69.8%, 5.9%) and EBMS, p-value = 0.114 and between infants on MF and EBF, p-value = 0.758.

**Table 2:** **HIV-free survival, HIV-infection and infants’ mortality according to the early infants’ feeding pattern**

	**Kaplan- Meier estimate at the 3rd month % (95CI)**	**P value = between EBF and EBMS**	**Kaplan- Meier estimate at the 18th month %(95CI)**	**P value = between EBF and EBMS**	**P value between HFS rates at 3rd month and 18th month**
		P value* = between EBF and MIXED		P value* = between EBF and MIXED	
		**P value^=between EBMS and MIXED**		**P value^=between EBMS and MIXED**	
**Cumulative overall HFS**	94.4%		87.1%		0.000
**HFS according to feeding pattern**					
EBF	97.4% (95.4 -99.4)	0.078	92.5% (89.26-95.74)	0.107	0.027
EBMS	99.1% (98.6- 99.6)	*0.000	86.2% (84.55-87.85)	*0.000	0.000
MF	75.7% (65.0 -86.4)	^0.000	69.8% (58.39-81.21)	^0.000	0.466
**HIV-infection**					
EBF	1.5% (0.004 -2.99)	0.027	7.0% (3.86-10.14)	0.237	0.007
EBMS	0.2% (0.014-0.414)	*0.000	4.3% (3.33-5.27)	*0.000	0.000
MF	1.26% (4.35-20.85)	^0.000	27.1% (16.05-38.15)	^0.000	0.047
**Death**					
EBF	0% (NA)	0.395	1.1% (−0.18-2.38)	0.567	0.142
EBMS	0.4% (0.092 – 0.703)	*0.002	2.1% (1.41-2.79)	*0.000	0.012
MF	4.9% (−0.47 -10.27)	^0.000	12.1 (3.99-20.21)	^0.000	0.157

**Legends**: HFS = HIV free survival; EBF = exclusive breast feeding; EBMS = exclusive breast milk substitute feeding; MF = mixed feeding; NA = not available. The rates of fall in HFS over the 3rd and the 18th month was compared by sample *t*-test for proportions between infants on: EBF and EBMS, p-value =0.002, t-statistics = 3.096; MF and EBMS, p-value = 0.114, t-statistics = 1.584; MF and EBF, p-value = 0.758, t-statistics = 0.309.

By the 3rd month, HIV-infection had been confirmed in 3 infants on EBF, 7 infants on MF but only in one infant on EBMS, with significant cumulative late-post-natal-HIV-infection rates of 1.5% (95% CI;0.004-2.99) in EBF, 0.2% (95CI;0.014-0.414) in EBMS and 12.6% (95%CI; 4.35-20.85) in MF groups. Pair-wise comparison revealed that significant differences occurred in HIV infection rates between EBF and EBMS babies (p-value: 0.027) and between EBF and MF babies (p-value; 0.000) and also between EBMS and MF infants (p-value; 0.000).

More HIV-infections occurred between the 3rd and the 18th month. This included 11 infants on EBF, 20 infants on EBMS and 13 infants on MF, with respective significant cumulative Post-natal-HIV-infection rates of 7.0% (95%CI; 3.86-10.14), 4.3% (95%CI; 3.33-5.27), and 27.1% (95CI;16.05-38.15). Significantly more HIV infections occurred among MF infants when compared with babies on EBF (p-value of 0.000) and among MF infants when compared with EBMS infants (p = 0.000) but not between EBF and EBMS infants (p = 0.237). Between the 3rd and the 18 months, the feeding pattern impacted significantly on HIV-infection rates for all the infant feeding groups; the EBF infants (p = 0.007), the EBMS infants (p = 0.000) and the MF infants (0.047).

The HIV-infection rates at 18 months were 3.73, 2.43 and 14.2 per child-year for the infants on EBF, EBMS and MF respectively.

By the 3 months of life, no death was recorded among EBF infants whereas 2 and 3 deaths were recorded among EBMS and MF infants respectively. All the two deaths in the EBMS infants were due to severe pneumonia and for the MF group, the 3 deaths resulted from diarrhoeal disease. Only one of the five infants that died was HIV-infected. Kaplan Meier estimate of death was only significant for EBMS infants at 0.4% (95%CI; 0.097-0.703). However, by the 18th month of life, mortality rates of 2.1% (95%CI; 1.41-2.79) occurred for EBMS infants and 12.1% (95% CI; 3.99-20.21) for MF infants. Two infants on EBF (diarrhoeal disease), 4 infants on EBMS (2 from disseminated tuberculosis, 2 from severe malaria) and 6 infants (2 from severe pneumonia and 4 from diarrhoeal disease) with MF had died. HIV-infection was diagnosed in 5 (4 among MF and 1 of EBF infants) of the 12 deaths (41.7%). More deaths occurred between the 3 rd month and 18th month for the EBMS infants, (p value 0.012).

**Figure 2:**
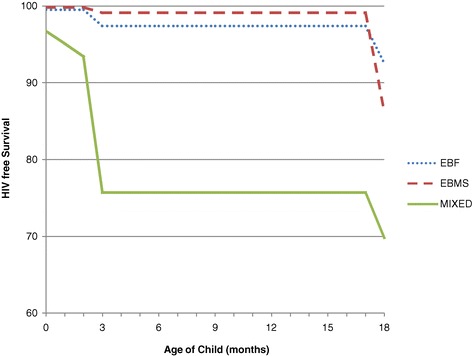
**HIV-free survival curves of the infants according to the early infant feeding pattern Legends: EBF: exclusive breast feeding; EBMS: exclusive breast milk substitute feeding; MF: mixed feeding.**

Figure 2 is the HIV-free survival curves of the infants according to the early infant feeding pattern.

### Cox proportional regression model for risk of HIV infection or death at 3 months and 18 months

Tables 3 and 4 reveal that in Cox regression multivariate analyses, only infants that had MF, those born at gestational age of ˂ 37 weeks, and those whose mothers had a high pre-delivery maternal viral load (10,000-99,999 copies/ml) were consistently associated with the risk of HIV infection or death at 3 months and 18 months. Mixed fed infants were 44 times more likely at 3 months and 5 times more likely at 18 months. Infants born at the gestational age of ˂ 37 weeks were 17 times more likely at the 3rd month and 8 times more likely at 18 months. Lastly, mothers with a high viral load in the range of 10,000-99,999 copies/ml were 10 times more likely and twice more likely to have died or contract the HIV infection at the 3rd month and the 18th month respectively.

**Table 3:** **Cox proportional regression model for risk of HIV infection or death at 3 months**

**Variable**	**HIV-infection or Death N (%)**	**Univariate analysis p-value**	**Multivariate aHR (95%CI)**	**analysis p-value**
Mode of feeding				
EBF (Ref)	3 (20.0)			
EBMS	3 (20.0)	0.209	0.368 (0.059 - 2.305)	0.286
MF	9 (60.0)	0.000	44.12 (5.574 - 349.214)	0.000
Birth weight (grams)				
≥2500 (Ref)	15 (100.0)			
˂2500	0 (0.0)	0.402	0.000 (0.000 - 0.000)	0.982
Gestational age				
(weeks)				
≥37 (Ref)	11 (78.3)			
˂37	4 (26.7)	0.000	17.00 (1.817 – 159.083)	0.013
Mode of delivery				
ELE C/S (Ref)	2 (13.3)			
Vaginal	12 (80.0)	0.000	0.023 (0.000 – 1.271)	0.065
EME C/S	1 (6.7)	0.043	0.083 (0.001 – 7.157)	0.273
ROM				
NO ROM (Ref)	2 (13.3)			
˂4 hours	11 (73.3)	0.000	0.962 (0.085 – 10.835)	0.975
≥4 hours	2 (13.3)	0.029	NA	NA
Vaginal tear				
No (Ref)	8 (53.3)			
Yes	7 (46.7)	0.031	2.309 (0.604 – 8.832)	0.221
Oral thrush				
No (Ref)	14 (93.3)			
Yes	1 (6.7)	0.0231	0.034 (0.000 – 2.952)	0.137
Oral sores				
No (Ref)	15 (100.0)			
Yes	0 (0.0)	0.719	0.000 (0.000 – 0.000)	0.990
Mothers’ age group in years				
15-24 (Ref)				
25-34	1 (6.7)			
35-39	10 (66.7)	0.319	NA	NA
≥40	3 (20.0)	0.178		
	1 (6.7)	0.155		
*Mothers’ CD4 count (Cells/mm^3^)				
≥500 (Ref)	3 (21.4)			
˂200	3 (21.4)	0.329	1.592 (0.295 – 8.588)	0.588
200-349	2 (14.3)	0.499	0.359 (0.055 – 2.355)	0.286
350-499	6 (42.9)	0.093	0.139 (0.020 – 0.964)	0.046
*Mothers’ viral load (Copies/ml)				
˂1000 (Ref)	7 (50.0)			
1000-9999	2 (14.3)	0.998	1.421 (0.233 – 8.669)	0.703
10,000-99,999	5 (35.7)	0.004	10.68 (1.559 – 73.174)	0.016
≥100,000	0 (0.0)	0.981	0.000 (0.000 – 0.000)	0.994
PMTCT intervention in the mother				
HAART	3 (20.0)			
ARV	12 (80.0)	0.036	0.639 (0.068 – 6.010)	0.695
*Mothers’ hemoglobin				
≥10 g/dl (Ref)	3 (20.0)			
˂10 g/dl	12 (80.0)	0.570	NA	NA
Parity				
1 (Ref)	3 (20.0)			
2-4	9 (60.0)	0.075	3.107 (0.628 – 15.369)	0.165
≥5	3 (20.0)	0.056	4.378 (0.415 – 46.155)	0.219
Breast problems reported or diagnosed at any time between birth and the 3rd month				
No (Ref)	14 (93.3)			
Yes	1 (6.7)	0.311	16.365 (1.359 – 197.091)	0.028
Mothers’ vital status at 3rd month				
Alive (Ref)	15 (100.0)			
Dead	0 (0.0)	0.814	0.000 (0.000 – 0.000)	0.997
Mothers’ level of education				
Some (Ref)	10 (66.7)			
None	5 33.3	0.274	NA	NA
Mothers’ marital status				
With a partner (Ref)	13 (86.7)	0.167	NA	NA
Without a partner	3 (13.3)			
Mothers/Family monthly income Minimum wage and above (Ref)				
Below minimum	1 (6.7)			
wage (~105 USD)	14 (93.3)	0.150	NA	NA

**Legends**: Ref = reference variable; EME C/S = emergency caesarean section; ELE C/S = elective caesarean section; EBF = exclusive breast feeding; EBMS = exclusive breast milk substitute feeding; MF = mixed feeding; ROM = rupture of membrane; PMTCT = prevention of mother to child transmission of HIV; HAART = highly active antiretroviral therapy; ARVs = antiretroviral prophylaxis; USD = United States dollars; * = pre-delivery values; NA = not available.

**Table 4:** **Cox proportional regression model for risk of HIV infection or death at 18 months**

	**HIV-infection or Death N (%)**	**Univariate analysis p-value**	**Multivariate aHR (95%CI)**	**Analysis p-value**
Mode of feeding				
EBF (Ref)	12 (23.5)			
EBMS	24 (47.1)	0.148	0.382 (0.175 – 0.832)	0.015
MF	15 (29.4)	0.000	5.182 (2.273 – 11.809)	0.000
Birth weight (grams)				
≥2500 (Ref)	47 (92.2)			
˂2500	4 (7.8)	0.568	0.808 (0.273 – 2.397)	0.701
Gestational age (weeks)				
≥37 (Ref)	41 (80.4)			
˂37	10 (19.6)	0.000	8.084 (2.921 – 22.375)	0.000
Mode of delivery				
ELE C/S (Ref)	2 (3.9)			
Vaginal	46 (90.2)	0.032	0.447 (0.045 – 4.452)	0.492
EME C/S	3 (5.9)	0.243	0.977 (0.078 – 12.298)	0.986
ROM				
NO ROM (Ref)	2 (3.9)			
˂4 hours	40 (78.4)	0.022	0.637 (0.224 – 1.811)	0.398
≥4 hours	9 (17.6)	0.706	NA	NA
Vaginal tear				
No (Ref)	33 (64.7)			
Yes	18 (35.3)	0.022	1.598 (0.820 – 3.113)	0.168
Oral thrush				
No (Ref)	49 (96.1)			
Yes	2 (3.9)	0.227	0.317 (0.043 – 2.338)	0.206
Oral sores				
No (Ref)	50 (98.0)			
Yes	1 (2.0)	0.857	3.015 (0.285 – 31.927)	0.359
Mothers’ age group in years				
15-24 (Ref)	8 (16.7)			
25-34	34 (66.7)	0.704	NA	NA
35-39	8 (15.7)	0.364		
≥40	1 (2.0)	0.837		
*Mothers’ CD4 count				
(Cells/mm^3^)				
≥500 (Ref)	8 (16.7)			
˂200	12 (25.0)	0.065	2.009 (0.683 – 5.904)	0.205
200-349	16 (33.3)	0.816	1.465 (0.596 – 3.600)	0.405
350-499	12 (25.0)	0.990	1.252 (0.544 – 2.883)	0.597
*Mothers’ viral load				
(Copies/ml)				
˂1000 (Ref)	25 (53.2)	0.265	1.468 (0.683 – 3.153)	0.325
1000-9999	10 (21.3)	0.001	2.526 (1.103 – 5.787)	0.028
10,000-99,999	11 (23.4)	0.864	0.617 (0.073 – 5.211)	0.658
≥100,000	1 (2.1)			
PMTCT intervention in the mother				
HAART	6 (11.8)			
ARVs	45 (88.2)	0.064	2.069 (0.784 – 5.460)	0.142
*Mothers’ hemoglobin				
≥10 g/dl (Ref)	13 (25.6)	0.058	0.755 (0.332 – 1.718)	0.530
10 g/dl	38 (74.5)			
Parity				
1 (Ref)	24 (47.1)			
2-4	22 (43.1)	0.942	NA	NA
≥5	5 (9.8)	0.760		
Breast problems reported or diagnosed at any time between birth and the 18th month				
No (Ref)	50 (98.0)			
Yes	1 (2.0)	0.834	1.437 (0.187 – 11.203)	0.729
Mothers’ vital status at 18 month				
Alive (Ref)	51 (100.0)			
Dead	0 (0.0)	0.707	0.000 (0.000 – 0.000)	0.973
Mothers’ level of education			NA	NA
Some (Ref)	38 (74.5)			
None	13 (25.5)	0.566		
Mothers’ marital status				
With a partner (Ref)	48 94.1	0.796	NA	NA
Without a partner	1 (5.9)			
Mothers/Family monthly income				
Minimum and above wage (Ref)	12 (23.5)			
Below minimum wage (~105 USD)	39 (76.5)	0.781	NA	NA

**Legends**: Ref = reference variable; EME C/S = emergency caesarean section; ELE C/S = elective caesarean section; EBF = exclusive breast feeding; EBMS = exclusive breast milk substitute feeding; MF = mixed feeding; ROM = rupture of membrane; PMTCT = prevention of mother to child transmission of HIV; HAART = highly active antiretroviral therapy; ARVs = antiretroviral prophylaxis; USD = United States dollars; * = pre-delivery values; NA = not available.

## Discussion

HIV Free survival provides greater insight into the efficiency of PMTCT programmes and helps influence choice of interventions. Among the 801 HIV uninfected infants at 6 weeks of life, the overall HFS was high at 94.4% at 3 months and 87.1% at the 18 months of age. The HFS rates for the infants on EBF were particularly impressive at the two time points even when ARVs were not administered to these infants for post exposure prophylaxis through breastfeeding period. We propose that, a higher HFS values are obtainable if ARVs had been administered to these infants through breastfeeding; although, the development of drug resistance and the prolonged exposure of HIV-uninfected children to unwarranted ARVs are contending issues. Infants that were mix fed had the lowest HFS rates at 3 months and 18 months and thus contributed adversely to the overall HFS rates seen.

The HFS rate of 92.5% in this study of HIV-exposed infants on EBF at 18 months is comparable to the reported HFS rates over 12–18 months in similar settings in other countries where breastfeeding took place for up to 6 months. Furthermore, the HFS rate of 92.5% was better than the HFS rates of: 84.7% in Kenya [20]; 84.9% in Botswana [21]; 86.4% in Tanzania [22] and 91.0% in South Africa [23]; but lower than the HFS rates of 96% in Cote d’Ivoire [24] and in Uganda [25].

In making comparison however, we are very cautious of conclusive generalization because of differences in the nature of our study and the designs of others [20-25].

The present study also reveals that infants who were mix fed had a greater likelihood of dying or contracting HIV infection at 3 months and at 18 months. In actual fact, infants on MF were responsible for most deaths, occurring majorly from diarrheal disease and severe pneumonia. HIV-infection was also documented in most of the dead mixed fed infants. Similar findings have been reported earlier [13,14].

In addition, infants born at the gestational age of ˂ 37 weeks were also found to be more at risk of HIV infection or death at the two time points. Along with previous studies [26,27], our study also supports the fact that preterm births may be associated with an increased risk of HIV infection.

Furthermore, infants of mothers with a high pre-delivery viral load (10,000-99,999 copies/ml) were more likely to have HIV infection or died at the 3rd month and at the 18th month. This finding is not unexpected as maternal viral load is a risk factor for post-natal HIV transmission; with the plasma viral loads correlating positively with the breast milk HIV viral loads and thus, a higher risk of HIV transmission through breastfeeding [16].

This study also revealed that at the 3rd month and 18th month of life, a respective late cumulative post-natal-HIV-transmission rates of 0.2% and 4.3% occurred among infants on EBMS. These findings have an important programmatic implication. It shows that, among mothers who reported ‘EBMS’, the HIV transmission seen in this group could only meant that these infants had been breastfed and were therefore mixed fed. It implies that even when infant formula and methods for sterilising water were provided free of cost, mothers still reverted to breastfeeding at some points in time and therefore efforts at EBMS feeding is not feasible in our setting and should be strongly discouraged.

Lastly, a higher and a significant drop off in HFS were seen among EBMS infants (from the 3rd to the 18th month) when compared to EBF. It shows that the apparent higher HFS rate that was seen earlier among our EBMS infants rapidly decayed over time and as early as the 18th month of follow-up. This significant decline further argues in favour of EBF over EBMS feeding.

### Limitations of study

Although we have tried to eliminate the possibility of an early post-natal- HIV transmission (occurring during pregnancy and labour) via exclusion of babies that were DNA/PCR positive at 6th week, on the other hand, a reasonable window of late post-natal -HIV transmission due to breast milk is actually possible from the 4th week. We do not have a control over this possibility. Firstly, this was a retrospective study and secondly, DNA/PCR screening is only routinely done at the 6th week and the 3rd month of life of the HIV-exposed infants in our ART programme protocol. Also, we were constrained to classifying the infants into the feeding patterns at the 6th week, rather than at the 6th month of the expected EBF or EBMS. We did this, so that we could take advantage of the knowledge of their HIV status at the 6th week as stated above. Also, the reported infants’ feeding pattern was by maternal recall and the possibility of bias is high. Regardless of these limitations, our study had a consistency in the feeding practice in accordance with a WHO guideline [10]. Secondly, the decision to breastfeed exclusively or not was not associated with some maternal and infants indicators of health as there were no significant differences (except for parity) in these variables between the feeding patterns. Rather, the choices in the feeding pattern (either EBF or EBMS) may have been informed by the pre-delivery counselling sessions that the mothers had during antenatal care.

## Conclusion

This study indicates that HIV-free survival rate was impressive and comparable for infants on EBF and EBMS at 3 months and at 18 months of life. It shows that MF, preterm deliveries and a high pre-delivery maternal viral load were consistently associated with a higher likelihood of infants’ death or HIV acquisition at 3 months and 18 months of follow-up. However, BMS feeding was associated with a significant drop off in HFS rate over time and babies on BMS reverted to MF. More also, in most African countries, when and where BMS is provided free, its sustainability depends on the generosity of the PMTCT Implementers which often, may not be predictable. For example, the free supply of EBMS offered to mothers in our programme came to an abrupt end in August 2011.

The 2010 WHO [28] recommendation still advocates that mothers known to be HIV-infected can give infant formula to their HIV uninfected infants or infants of an unknown status, when AFASS are met; our study has clearly shown that even when these infant formula are provided free of cost and efforts at safety preparation of this infant formula attempted, mothers still mixed fed their babies by engaging in breastfeeding. We therefore strongly recommend that EBF is the most feasible, most practicable and the safest mode of infant feeding within the context of HIV infection in our setting.
